# VARIATION IN STATE APPROACHES TO DEMENTIA CARE TRAINING IN ASSISTED LIVING

**DOI:** 10.1093/geroni/igae098.1658

**Published:** 2024-12-31

**Authors:** Paula Carder, Lindsey Smith, Sarah Dys, Eric Jutkowitz, Kali Thomas

**Affiliations:** OHSU-PSU School of Public Health, Portland, Oregon, United States; Oregon Health & Science University, Portland, Oregon, United States; Portland State University, Portland, Oregon, United States; Brown University, Brown University, Rhode Island, United States; Johns Hopkins University, Baltimore, Maryland, United States

## Abstract

Assisted living (AL) communities increasingly serve individuals with Alzheimer’s disease and related disorders (ADRD); an estimated 50% of AL residents have ADRD, and more AL have designated memory care than do nursing homes. State AL regulations vary on almost every provision that affects resident safety and quality, including staffing levels, building design, safety features, admission and discharge criteria, and social-recreational programming. Nearly every state requires that direct care workers receive training in dementia care. Our prior research described the presence of within-state variation in memory care regulations, with the majority of states requiring such training only in designated “memory care” settings rather than all AL licenses (Smith et al., 2022). This paper extends that research by examining staff dementia care training requirements in detail, including the number of training hours, specificity of dementia care topics, and the presence of social aspects of care (e.g., family involvement, person-centered care). The 43 states that require direct care workers to receive training in dementia care have a total of 93 license types, with some applied to settings that are designated as “memory care.” Of the 43 states and 93 licenses, 23 states and 34 licenses specify the number of training hours that direct care workers must receive in the first year, ranging from 2 to 75 hours (average 15.7). Examples of highly specific staff training requirements are provided as a resource for states and AL professionals, and to inform consumers who might expect that all memory care settings have similar requirements.

